# OTUB1 inhibits breast cancer by non‐canonically stabilizing CCN6

**DOI:** 10.1002/ctm2.1385

**Published:** 2023-08-22

**Authors:** Ying Zhao, Jing Ruan, Zhongding Li, Xian Su, Kangmin Chen, Yimin Lin, Yuepiao Cai, Peng Wang, Baohua Liu, Dirk Schlüter, Guang Liang, Xu Wang

**Affiliations:** ^1^ Chemical Biology Research Center School of Pharmaceutical Sciences Wenzhou Medical University Wenzhou China; ^2^ Oujiang Laboratory (Zhejiang Lab for Regenerative Medicine, Vision and Brain Health) Department of Neurological Rehabilitation The Second Affiliated Hospital and Yuying Children's Hospital of Wenzhou Medical University Wenzhou China; ^3^ Department of Pathology The First Affiliated Hospital Wenzhou Medical University Wenzhou China; ^4^ School of Pharmaceutical Sciences Wenzhou Medical University Wenzhou China; ^5^ Institute of Medical Microbiology and Hospital Epidemiology Hannover Medical School Hannover Germany; ^6^ Cluster of Excellence RESIST (EXC 2155) Hannover Medical School Hannover Germany; ^7^ School of Pharmaceutical Sciences Hangzhou Medical College Hangzhou China

**Keywords:** breast cancer, CCN6, OTUB1, protein degradation, ubiquitination

## Abstract

**Background:**

CCN6 is a matricellular protein that critically regulates the tumourigenesis and progression of breast cancer. Although the tumour‐suppressive function of CCN6 has been extensively studied, molecular mechanisms regulating protein levels of CCN6 remain largely unclear. This study aims to investigate the regulation of CCN6 by ubiquitination and deubiquitinating enzymes (DUBs) in breast cancer.

**Methods:**

A screening assay was performed to identify OTUB1 as the DUB for CCN6. Various biochemical methods were applied to elucidate the molecular mechanism of OTUB1 in the regulation of CCN6. The role of OTUB1–CCN6 interaction in breast cancer was studied with cell experiments and the allograft model. The correlation of OTUB1 and CCN6 in human breast cancer was determined by immunohistochemistry and Western blot.

**Results:**

We found that CCN6 protein levels were controlled by the ubiquitin–proteasome system. The K48 ubiquitination and degradation of CCN6 was inhibited by OTUB1, which directly interacted with CCN6 through its linker domain. Furthermore, OTUB1 inhibited the ubiquitination of CCN6 in a non‐canonical manner. Deletion of OTUB1, concomitant with reduced CCN6 abundance, increased the migration, proliferation and viability of breast cancer cells. Supplementation of CCN6 abolished the effect of OTUB1 deletion on breast cancer. Importantly, OTUB1 expression was downregulated in human breast cancer and positively correlated with CCN6 levels.

**Conclusion:**

This study identified OTUB1 as a novel regulator of CCN6 in breast cancer.

## Graphical abstract

• OTUB1 interacts with CCN6 through the linker domain and stabilizes CCN6 protein by reducing K48 ubiquitination.

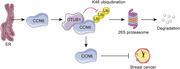

## 1 INTRODUCTION

With more than 2.3 million new cases per year, breast cancer has become the most commonly diagnosed cancer in 2020, accounting for 11.7% of total cancer cases.^1^ Despite substantial progresses in the therapy of breast cancer that have been achieved in the past years, breast cancer is still a leading cause of cancer‐related death in women worldwide.^2^, ^3^ Unraveling the mechanisms underlying the pathogenesis and development of breast cancer is beneficial for the development of effective diagnostic and therapeutic approaches for this life‐threatening disease.

The matricellular protein CCN6 has emerged as a key suppressor in breast cancer.^4^, ^5^, ^6^ CCN6, also known as WNT‐inducible signalling pathway protein 3 (WISP3), is a protein belonging to the CCN family, which consists of six cystine rich, glycosylated, secreted proteins.^7^ The CCN family members, CCN1‐6, are evolutionarily conserved proteins that can regulate various pathophysiological processes, in particular, cancer.^7^, ^8^, ^9^ Both the mRNA and protein of CCN6 are expressed in normal breast epithelia. However, frameshift mutations in the *Ccn6* gene have been detected in a subset of human metaplastic breast carcinomas.^10^ Moreover, CCN6 expression is markedly reduced in aggressive breast cancer types, including metaplastic carcinomas and inflammatory breast cancer.^5^, ^11^ Importantly, mice with mammary epithelial cell‐specific deletion of CCN6 developed invasive mammary carcinomas resembling human metaplastic breast cancer, providing a direct evidence that CCN6 is a tumour suppressor for breast cancer.^5^

Overexpression of CCN6 protein in aggressive breast cancer cells with low endogenous CCN6 abundance diminished their aggressive phenotypes, indicating that increasing the protein expression of CCN6 is favourable for breast cancer treatment.^6^ In addition to synthesis, secretion (for secreted proteins) and degradation are main factors regulating protein levels. Of note, 80%−90% of cellular proteolysis is mediated by the ubiquitin–proteasome system (UPS), with the rest mainly carried out by the autophagy‐lysosomal pathway (ALP).^12^ The UPS induces the 26S proteasome‐dependent degradation of proteins labelled with ubiquitination, a post‐translational modification involving the attachment of one or more ubiquitin molecules. Besides, ubiquitination can also induce protein degradation through the ALP.^13^ Therefore, ubiquitination serves as a crucial signal for proteolysis mediated by both the UPS and ALP. Noteworthy, ubiquitination is a reversible process that is sequentially catalysed by E1, E2 and E3 ubiquitinating enzymes and inhibited by deubiquitinating enzymes (DUBs).^14^, ^15^ Among the ∼100 DUBs, several members, such as A20, USP43, OTUD1, OTUD3 and USP13, have been identified as key regulators in breast cancer.^16^, ^17^, ^18^, ^19^, ^20^ For example, OTUD3 and USP13 can deubiquitinate and stabilize the tumour suppressor PTEN, thereby playing a suppressing role in breast cancer.^19^, ^20^

To date, a full comprehension of the mechanisms regulating CCN6 protein levels is still out of reach. Particularly, it is unknown whether CCN6 protein levels are regulated by protein degradation. In this study, we found that CCN6 protein turnover is regulated by the UPS. We also identified OTU domain, ubiquitin aldehyde binding 1 (OTUB1) as the DUB deubiquitinating CCN6. OTUB1 directly interacted with CCN6 through its linker domain to reduce the K48 polyubiquitination and degradation of CCN6 in a non‐canonical way. In addition, OTUB1 suppressed the aggressive phenotypes of breast cancer both in vitro and in vivo by elevating CCN6 levels. In human breast cancer samples, OTUB1 expression levels were positively correlated with that of CCN6. Together, our results reveal a novel and critical role of OTUB1 in regulating CCN6 stability and breast cancer.

## 2 MATERIALS AND METHODS

### 2.1 Cell culture

The mouse breast cancer cell line 4T1, as well as human breast cancer cell lines MDA‐MB‐231 and BT549, was purchased from Shanghai Institute of Biochemistry and Cell Biology (Shanghai, China). Cells were cultured in RPMI 1640 or DMEM medium (Gibco) supplemented with 1% penicillin/streptomycin (Invitrogen) and 10% foetal bovine serum (FBS; Gibco) in an incubator maintained at 37 °C with 5% CO_2_.

### 2.2 RNA interference

Cultured cells were transfected with siRNA targeting *OTUB1* (sense: GCGACUCCGAAGGUGUUAATT) or *Hrd1* (sense: CTGCCATGCTTCAAATCAA) with Lipofectamine RNAiMAX according to the producer's protocols (Invitrogen). Forty‐eight hours later, transfected cells were used for further analysis.

### 2.3 Generation of OTUB1 knockout cell lines

*OTUB1* gRNA (sequence: AGAATCCTCTGGTGTCAGAG) was cloned into lentiCas9‐Blast (Plasmid #52962, Addgene, USA). For lentivirus package, lentiCas9‐Blast‐OTUB1 gRNA plasmids were transfected into 293T cells together with pMD2.G and psPAX2 plasmids. The medium containing lentivirus was harvested at 48 h after transfection. Then, lentivirus was added to 4T1 cell cultures with 8 μg/mL polybrene (Sigma‐Aldrich). Seventy‐two hours after transduction, 4 μg/mL blasticidin (Yeasen Biotechnology, Shanghai) was used to select single‐cell clones. The ablation efficiency was confirmed by Western blot.

### 2.4 Wound healing assay

4T1 cells were added to 6‐well plates at a density of half a million cells per well and grown to confluence. Then, a sterile 10 μL micropipette tip was applied to make a linear wound. Thereafter, cells were rinsed with PBS to wash off detached cells and debris. Wound closure was observed and recorded at indicated time points.

### 2.5 Cell proliferation assay

4T1 cells were seeded in 12‐well plates at a density of 100,000 cells per well. Then, cells were cultured in RPMI 1640 medium containing 1% penicillin/streptomycin and 10% FBS for the indicated times. Cells were fixed in 4% paraformaldehyde followed by staining with the crystal violet staining solution (Beyotime Biotechnology, Shanghai).

### 2.6 MTT assay

4T1 cells were added to 96‐well plates at a density of 5000 cells per well and then cultured for the indicated times. MTT solution (Solarbio, Beijing) at a final concentration of 1 mg/mL was added to each well. After incubation for 4 h, the supernatant was discarded and 150 μL of DMSO (Solarbio, Beijing) was added to each well. The absorbance was determined at the wavelength of 490 nm.

### 2.7 Western blot

Tumour tissues and cultured cells were lysed in RIPA lysis buffer with PMSF and protease inhibitor cocktail (Cell Signaling Technology). Insoluble components were removed by centrifugation at 12,000 × *g* at 4°C for 10 min. Supernatants were collected and denatured in SDS loading buffer at 98°C for 10 min. After denaturing, samples were separated by SDS‐PAGE and then transferred to PVDF membranes. Subsequently, immunoblotting was carried out with antibodies against CCN6 (Novus Biologicals, NBP2‐93872) 1:2000, OTUB1 (Novus Biologicals, NBP1‐49934) 1:2000, GAPDH (Bioworld, MB001) 1:10,000, His‐Tag (Proteintech, 66005‐1‐IG) 1:1000, GFP‐Tag (OriGene, TA150041) 1:5000, HA‐Tag (Proteintech, 51064‐2‐AP) 1:1000 and FLAG‐Tag (Proteintech, 20543‐1‐AP) 1: 1000, respectively. An ECL Plus Kit (GE Healthcare) was used to develop the blots and images were taken by the ChemiDoc^™^ XRS+ Imaging System (BIO‐RAD). Uncropped original Western blot images were included in the Supporting Information.

### 2.8 Immunoprecipitation

Whole‐cell lysates were generated as described before in ‘Western blot’. Sepharose beads (Beyotime Biotechnology) were added to cell lysates and incubated under continuous rotation at 4°C for 2 h to remove unspecific bead‐binding proteins. Then, the samples were centrifugated at 12,000 × *g* at 4°C for 5 min to remove beads. After that, anti‐OTUB1 (2 μg/mg lysate) or anti‐FLAG (1 μg/mg lysate) antibodies were added to precleared lysates and incubated under continuous rotation at 4°C overnight. Sepharose beads were subsequently added to capture the immunocomplexes. After a 2 h incubation at 4°C, the beads were rinsed five times with ice‐cold PBS by centrifugation at 12,000  × *g* for 15 s. The beads were resuspended in 2 × SDS loading buffer and denatured at 98°C for 10 min. After denaturing, the samples were centrifuged at 12,000 × *g* for 1 min, and the supernatant was harvested for Western blot.

### 2.9 In vitro interaction assay

The purified His‐OTUB1 protein (HUABIO, Hangzhou) was incubated with 4T1 cell lysates at 4°C overnight. After incubation, His‐OTUB1 and interacting proteins were harvested with anti‐His antibody (1 μg/mg lysate) as described in Section 2.8. Western blot was applied to analyse the presence of OTUB1 and CCN6.

### 2.10 Cycloheximide chase experiment

CCN6‐FLAG plasmids were transfected into 4T1 cells with Lipofectamine 3000 (Invitrogen) according to the producer's instructions. After 24 h, cells were treated with 100 μg/mL cycloheximide (Merck) for the indicated times. Thereafter, cells were collected for Western blot analysis.

### 2.11 In vitro deubiquitination assay

Equal amount of GFP (OriGene, PS100010), OTUB1‐GFP (OriGene, MG203606) or CCN6‐FLAG (OriGene, MR223941) + K48 Ub‐HA (Addgene, #17605) plasmids were transfected into 4T1 cells with Lipofectamine 3000 (1 μg plasmid/1 million cells). Except for the K48 residue, all other lysines were mutated to arginines in the K48 Ub‐HA plasmid. Similarly, in the K63 Ub‐HA plasmid, all lysines except for K63 were mutated to arginines. After 24 h, GFP, OTUB1‐GFP and K48 ubiquitinated CCN6 were purified from cell lysates by immunoprecipitation. Immunoprecipitated samples were washed sequentially with PBS and deubiquitination buffer (5 mM MgCl_2_, 5% glycerol, 50 mM Tris‐HCl, 2 mM ATP‐Na_2_ and 2 mM DTT). Then, K48 ubiquitinated CCN6 was incubated with GFP or OTUB1‐GFP in the deubiquitination buffer at 37°C for 2 h. Thereafter, samples were analysed by Western blot.

### 2.12 Endoplasmic reticulum extraction

An endoplasmic reticulum extraction kit (Bestbio, Nanjing, China) was used to extract endoplasmic reticulum from 4T1 cells according to the producer's instructions. Endoplasmic reticulum and non‐endoplasmic reticulum fractions were lysed in complete RIPA lysis buffer, followed by Western blot analysis.

### 2.13 Quantitative RT‐PCR

Total mRNA was extracted from cultured cells or tumour tissues with the RNeasy Kit (Qiagen) according to the producer's instructions. The SuperScript reverse transcriptase kit (Invitrogen) was applied to generate cDNA from mRNA. Quantitative RT‐PCR for *CCN6* (forward primer: 5ʹ‐TGTGGCAGTTGGATGTGAGT‐3ʹ, reverse primer: 5ʹ‐CCCGGTTAGAAATTCCCA TT‐3ʹ) and *β‐actin* (forward primer: 5ʹ‐GGTCATCACTATTGGCAACG‐3ʹ, reverse primer: 5ʹ‐ACGGATGTCAACGTCACACT‐3ʹ) was performed on a QuantStudio Real‐Time PCR System (Thermo Fisher Scientific). *CCN6* expression was normalized to *β‐actin* and changes of *CCN6* were calculated through relative quantification.

### 2.14 Transwell migration assay

Uncoated polycarbonate membranes with 8 μm pores were used for transwell migration assays. 4T1 cells in 200 μL of serum‐free RPMI 1640 medium were added to a 24‐well migration chamber, and 600 μL of RPMI 1640 medium containing 10% FBS was added to the lower chamber. After incubation in a cell incubator for 24 h, cells migrated to the lower chamber were fixed and then stained with crystal violet staining solution. Images were recorded and cells were counted in five random fields per insert.

### 2.15 In vivo allograft model

Female BALB/C‐Nude mice (6‐ to 8‐week old) were obtained from Vital River Laboratories (Beijing, China). Ten million 4T1 cells in 150 μL of PBS were injected subcutaneously into each mouse. On days 5, 7 and 9 after tumour inoculation, tumour volume (*V*) was measured and calculated as *V* = 0.5 × length × width.^2^ Then, mice were sacrificed on day 9 after tumour inoculation, and tumours were removed, weighed, photographed and further analysed. Animal care and experiments were carried out under SPF conditions in the Laboratory Animal Centre, Wenzhou Medical University. The animal experiments were approved by the Animal Management and Ethics Committee of Wenzhou Medical University (Approval number: wydw2022‐0300).

### 2.16 Clinical samples

Tumour biopsy samples were obtained from The First Affiliated Hospital of Wenzhou Medical University. Fresh tumour specimens and paired tumour–adjacent normal tissues were used for analysis. The patients did not receive radiotherapy or chemotherapy before the operation and they did not have other malignant tumours. This study was approved by the Ethics Committee in Clinical Research (ECCR) of The First Affiliated Hospital of Wenzhou Medical University for the use of clinical biopsy specimens (Approval number: KY2022‐R091) and informed consent was obtained from patients. All aspects of the study followed the Declaration of Helsinki of 1975, revised in 2008.

### 2.17 Immunohistochemistry

Tumour or clinical samples were fixed in 4% paraformaldehyde for 48 h and then embedded in paraffin for immunohistochemistry (IHC). Allografted tumour samples were stained with Ki‐67 to assess tumour cell proliferation. Clinical specimens were stained for OTUB1 and CCN6. In short, slides were boiled in sodium citrate buffer for epitope retrieval. Then, endogenous peroxidase activity was quenched with 3% H_2_O_2_. After blocking with 5% bovine serum albumin, different slices were incubated with primary antibodies against Ki‐67 (Abcam, ab16667, 1:200), OTUB1 (Novus Biologicals, NBP1‐49934, 1:200) and CCN6 (Novus Biologicals, NBP2‐93872, 1:100) at 4°C overnight. Then, horseradish peroxidase‐conjugated secondary antibodies (Beyotime Biotechnology, 1:200) and 3,3′‐diaminobenzidine (ZSGB‐BIO, Beijing) were used for signal detection.

### 2.18 Statistics

Statistical difference was analysed with the GraphPad Prism 8 software (GraphPad). The two‐tailed Student's *t*‐test was applied to compare data between two groups, and the one‐way ANOVA test was applied to analyse more than two groups of data. *p* values < .05 were considered significant. Experiments were performed at least twice.

## 3 RESULT

### 3.1 CCN6 protein expression is positively regulated by OTUB1

**FIGURE 1:**
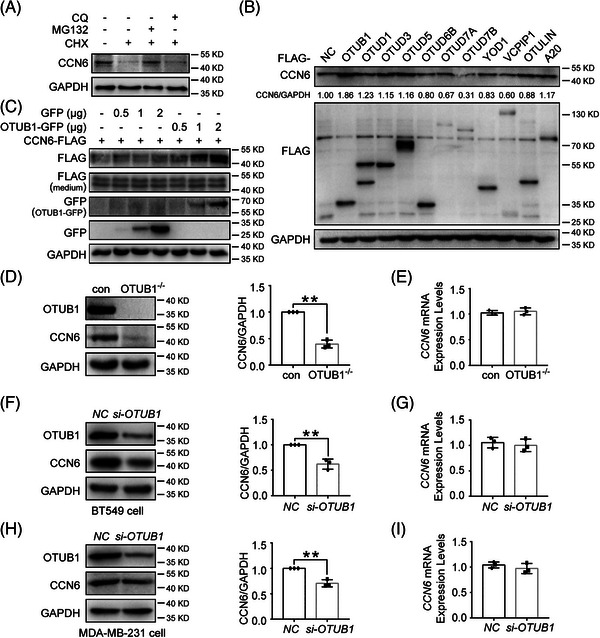
OTUB1 increases the protein expression of CCN6. (A) 4T1 cells were left untreated or treated with cycloheximide (CHX, 100 μg/mL), MG132 (10 μM) and CQ (50 μM), either alone or in combination as indicated, for 9 h. Thereafter, CCN6 protein abundance was analysed by Western blot. (B) Plasmids encoding FLAG‐tagged OTU family DUBs were transfected into 4T1 cells. Twenty‐four hours after transfection, endogenous CCN6 protein abundance was analysed by Western blot. (C) 4T1 cells were transfected with CCN6‐FLAG plasmids together with various amounts of GFP vector plasmids or OTUB1‐GFP plasmids. Twenty‐four hours after transfection, whole‐cell lysates were analysed by Western blot. (D) The protein abundance of OTUB1 and CCN6 in control and OTUB1^−/−^ 4T1 cells was analysed by Western blot (left panel). The right panel shows the relative protein levels of CCN6 normalized to GAPDH (*n* = 3 for both groups) (mean ± SEM, *p < .05). (E) The relative mRNA levels of CCN6 in control and OTUB1^−/−^ 4T1 cells were analysed by qRT‐PCR (*n* = 3 for both groups). (F) BT549 cells were transfected with nonsense siRNA or *OTUB1* siRNA for 48 h. Thereafter, whole‐cell lysates were analysed by Western blot to detect OTUB1 and CCN6 levels (left panel). The right panel shows the relative protein levels of CCN6 normalized to GAPDH (*n* = 3 for both groups) (mean ± SEM, *p < .05). (G) BT549 cells were transfected with nonsense siRNA or *OTUB1* siRNA for 48 h. The relative mRNA levels of *CCN6* were then analysed by qRT‐PCR (*n* = 3 for both groups). (H) MDA‐MB‐231 cells were transfected with nonsense siRNA or *OTUB1* siRNA for 48 h. Thereafter, whole‐cell lysates were analysed by Western blot to detect OTUB1 and CCN6 levels (left panel). The right panel shows the relative protein levels of CCN6 normalized to GAPDH (*n* = 3 for both groups) (mean ± SEM, *p < .05). (I) MDA‐MB‐231 cells were transfected with nonsense siRNA or *OTUB1* siRNA for 48 h. The relative mRNA levels of *CCN6* were then analysed by qRT‐PCR (*n* = 3 for both groups).

CCN6 has been proven to be a suppressor in breast cancer and CCN6 protein is downregulated in human breast cancer tissues.^5^, ^11^ However, it remains unknown whether and how CCN6 protein is degraded. Blocking new protein synthesis with cycloheximide strongly reduced CCN6 protein levels in 4T1 mouse breast cancer cells (Figure 1A and Figure S1), indicating that CCN6 protein levels are tightly regulated by protein degradation. In addition, the proteasome inhibitor MG132, rather than the lysosome inhibitor chloroquine (CQ), prevented the degradation of CCN6 (Figure 1A and Figure S1), showing that CCN6 degradation is mainly mediated by proteasomes. Similar result was obtained in the normal breast MCF10A cell line (Figure S2). Since proteasomes degrade ubiquitinated proteins and ubiquitination is inhibited by DUBs, we sought to identify DUBs that can deubiquitinate and stabilize CCN6. In the screening experiment, we found that overexpression of OTUB1, a DUB of the ovarian tumour proteases (OTU) subfamily, markedly increased the protein abundance of CCN6 in 4T1 cells (Figure 1B). To confirm this result, we transfected 4T1 cells with increasing amounts of plasmids encoding GFP‐tagged OTUB1 and found that OTUB1 increased CCN6 protein levels in cells in a dose‐dependent manner (Figure 1C). Since CCN6 is a secreted protein, we analysed CCN6 levels in the culture medium. Overexpression of OTUB1 did not change the protein concentration of secreted CCN6 in the culture medium, ruling out the possibility that the increased CCN6 protein levels in OTUB1‐overexpressing 4T1 cells were caused by reduced secretion (Figure 1C). Of note, two bands were constantly detected for overexpressed FLAG‐tagged CCN6, possibly due to an N‐terminal truncation. To explore the function of OTUB1 in 4T1 cells, we generated OTUB1^−/−^ 4T1 cells using the CRISPR/Cas9 technology (Figure 1D). Consistent with Figure 1B,C, ablation of OTUB1 significantly reduced CCN6 protein expression in 4T1 cells (Figure 1D). Since CCN6 is a secreted protein and ubiquitination occurs mainly in the cytosol, we hypothesized that OTUB1 affected the ubiquitination‐dependent degradation of CCN6 through the endoplasmic reticulum associated protein degradation (ERAD) process. To test this idea, we extracted endoplasmic reticulum and non‐endoplasmic reticulum fractions from control and OTUB1^−/−^ 4T1 cells. We found that OTUB1 ablation decreased CCN6 protein abundance only in the non‐endoplasmic reticulum fraction (Figure S3A). Protein retrotranslocation and ERAD is critically mediated by the ubiquitin ligase Hrd1.^21^ Knockdown of Hrd1 blunted the difference in CCN6 protein abundance between control and OTUB1^−/−^ 4T1 cells (Figure S3B). These results indicate that OTUB1 regulates the ubiquitination and degradation of CCN6 through the ERAD. Of note, mRNA levels of *CCN6* were not affected by OTUB1 deletion (Figure 1E). To substantiate this finding in human breast cancer cells, we transfected MDA‐MB‐231 and BT549 cells with *OTUB1* siRNA. Similar to findings obtained with 4T1 cells, knockdown of OTUB1 significantly reduced CCN6 protein levels in these human breast cancer cells, but did not influence mRNA levels of *CCN6* (Figure 1F–I). Together, these results show that the protein abundance of CCN6 is enhanced by OTUB1, which possibly inhibits CCN6 protein degradation rather than augmenting its production.

### 3.2 OTUB1 stabilizes CCN6 protein by reducing K48 ubiquitination

**FIGURE 2:**
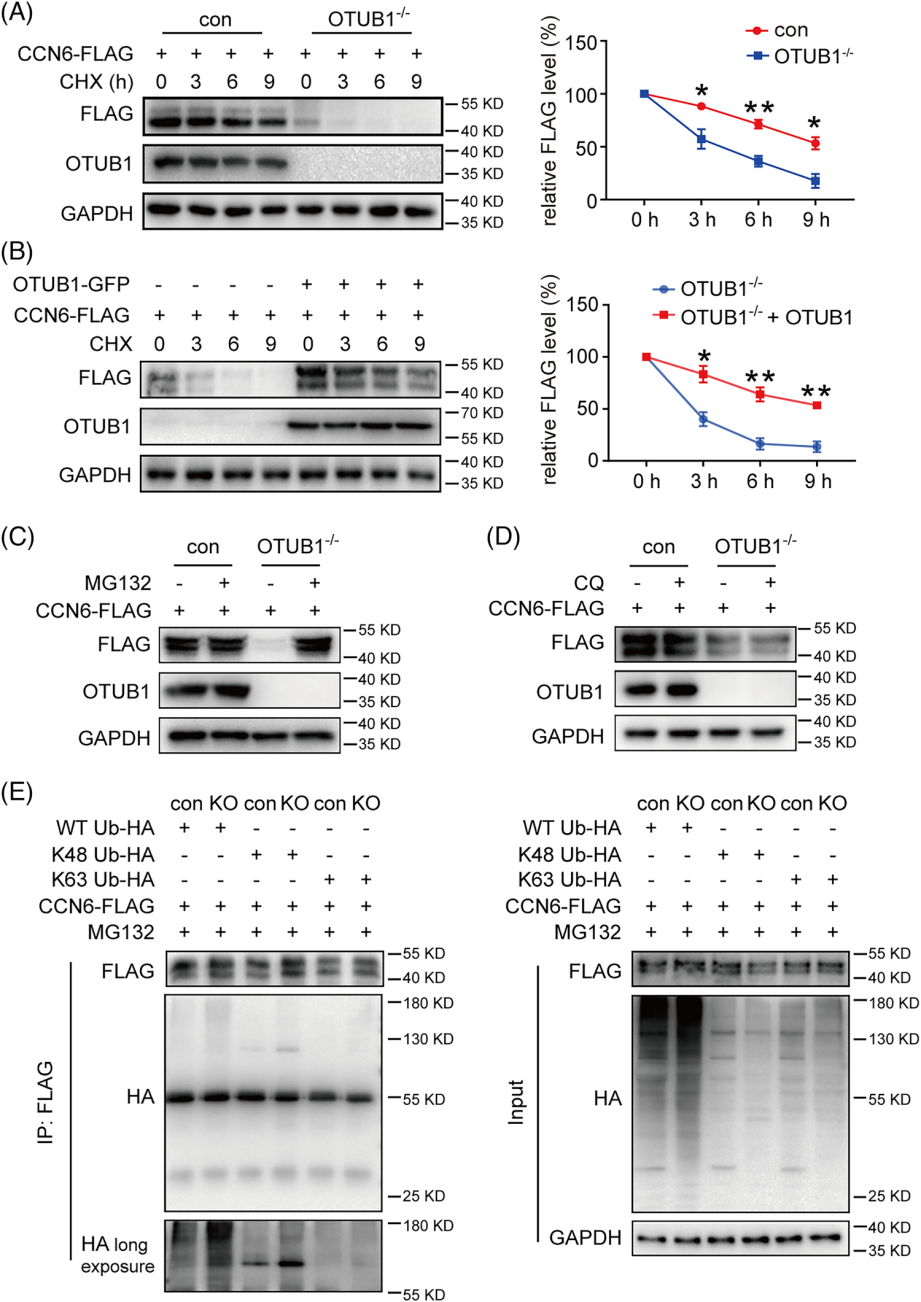
OTUB1 deubiquitinates and stabilizes CCN6 protein. (A) Control and OTUB1^−/−^ 4T1 cells were transfected with CCN6‐FLAG plasmids. Twenty‐four hours after transfection, cells were treated with cycloheximide (100 μg/mL) for the indicated times. Protein levels of CCN6 and OTUB1 were analysed by Western blot (left panel). The right panel shows the relative protein expression of CCN6 normalized to GAPDH (*n* = 3 for both groups) (mean ± SEM, *p < .05). (B) OTUB1^−/−^ 4T1 cells were transfected with CCN6‐FLAG plasmids or co‐transfected with CCN6‐FLAG and OTUB1‐GFP plasmids. Twenty‐four hours after transfection, cells were treated with cycloheximide (100 μg/mL) for the indicated times. Protein levels of CCN6 and OTUB1 were analysed by Western blot (left panel). The right panel shows the relative protein expression of CCN6 normalized to GAPDH (*n* = 3 for both groups) (mean ± SEM, *p < .05). Control and OTUB1^−/−^ 4T1 cells were transfected with CCN6‐FLAG plasmids. Twenty‐four hours after transfection, cells were treated with (C) 10 μM MG132 or (D) 50 μM CQ for 0 and 6 h. Thereafter, whole‐cell lysates were analysed by Western blot. (E) 4T1 cells were transfected with indicated plasmids. Twenty‐four hours after transfection, cells were treated with MG132 (10 μM) for 6 h before lysation. Proteins were immunoprecipitated with anti‐FLAG antibody and analysed by Western blot with indicated antibodies.

Since CCN6 protein levels are tightly regulated by degradation, we analysed the impact of OTUB1 on CCN6 protein degradation. Upon treatment with cycloheximide, the ablation of OTUB1 significantly accelerated the degradation of CCN6 in 4T1 cells (Figure 2A). Consistently, overexpression of OTUB1 in OTUB1^−/−^ 4T1 cells inhibited CCN6 degradation (Figure 2B), showing that OTUB1 increases CCN6 protein levels by inhibiting its degradation. To clarify the mechanism by which OTUB1 blocked CCN6 degradation, control and OTUB1^−/−^ 4T1 cells were treated with MG132 or CQ. As shown in Figure 2C,D, inhibition of proteasome rather than lysosome blunted the difference in CCN6 protein levels between control and OTUB1^−/−^ 4T1 cells. Similarly, we confirmed this finding in human breast cancer cells MDA‐MB‐231 and BT549 (Figure S4). This suggests that OTUB1 inhibits proteasome‐dependent degradation of CCN6. Given that K48‐specific polyubiquitination leads to proteasome‐mediated protein degradation and that OTUB1 is a DUB that specifically cleaves K48‐linked polyubiquitin chains, it is probable that OTUB1 inhibits CCN6 degradation by reducing its K48 ubiquitination. Deletion of OTUB1 increased the total and K48 ubiquitination of CCN6 (Figure 2E and Figure S5A). In contrast, OTUB1 ablation had no obvious influence on K63 ubiquitination and other ubiquitination types (Figure 2E and Figure S5B). Collectively, these data show that OTUB1 increases the protein abundance of CCN6 by reducing its K48 ubiquitination and degradation.

### 3.3 The linker domain of OTUB1 is required for the interaction with CCN6

**FIGURE 3:**
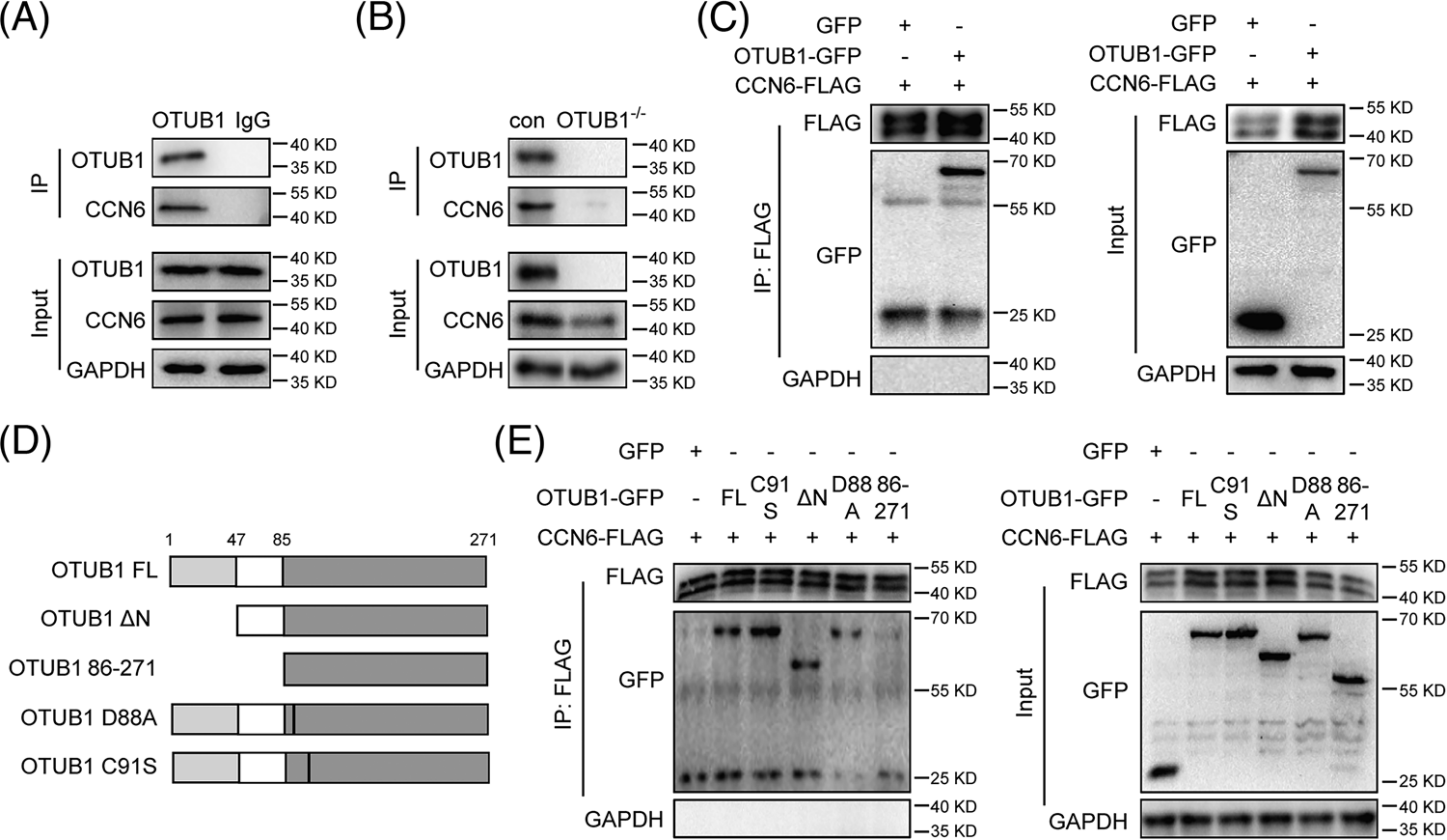
OTUB1 physically binds with CCN6. (A, B) 4T1 cells were treated with MG132 (10 μM) for 6 h before lysation. Immunoprecipitation was performed with anti‐OTUB1 antibody and rabbit IgG. The immunoprecipitates were analysed by Western blot for OTUB1, CCN6 and GAPDH. (C) 4T1 cells were co‐transfected with CCN6‐FLAG plasmids and GFP vector plasmids or OTUB1‐GFP plasmids. Twenty‐four hours after transfection, proteins were collected from whole‐cell lysates by immunoprecipitation with anti‐FLAG antibody and analysed by Western blot for FLAG, GFP and GAPDH. (D) A schematic picture depicting OTUB1 domains and different mutants. (E) 4T1 cells were transfected with indicated plasmids. Twenty‐four hours after transfection, proteins were immunoprecipitated from whole‐cell lysates with anti‐FLAG antibody and analysed with Western blot for FLAG, GFP and GAPDH.

We then explored whether OTUB1 regulates CCN6 stability by direct interaction. Endogenous OTUB1 and its interacting proteins were harvested from whole‐cell lysates of 4T1 cells by co‐immunoprecipitation (co‐IP) with anti‐OTUB1 antibody. CCN6 was co‐precipitated from 4T1 cell lysates together with OTUB1 by anti‐OTUB1 antibody (Figure 3A,B), suggesting an interaction between endogenous OTUB1 and CCN6. To consolidate this finding, we overexpressed exogenous CCN6‐ FLAG and OTUB1‐GFP or GFP in 4T1 cells and performed co‐IP with anti‐FLAG antibody. OTUB1‐GFP, but not GFP, was detected in the immunocomplex, showing that CCN6 directly interacts with OTUB1 (Figure 3C). Besides, in the in vitro interaction assay, recombinant His‐OTUB1 was found to interact with CCN6 (Figure S6). At both endogenous and exogenous levels, these results collectively reveal that OTUB1 is a bona fide binding protein of CCN6.

Structurally, OTUB1 is a 271‐aa protein with an N‐terminal UBA domain (1‐47 aa), a linker domain (48‐85 aa) and a C‐terminal OTU domain (86‐271 aa). Noteworthily, the D88 and C91 residues in the OTU domain are critical for the deubiquitinating activity of OTUB1. Molecular docking was performed to predict the interaction between OTUB1 and CCN6. Based on the crystal structure of OTUB1 (PDBID:4DDG) and the high similarity between OTUB1‐interacting region of polyubiquitin‐C and a region of CCN6, hydrogen bonds were predicted to form between CCN6 and the linker domain of OTUB1 (Figure S7). Therefore, it is possible that OTUB1 interacts with CCN6 through its linker domain. To determine the domains or residues of OTUB1 that are required to interact with CCN6, we generated plasmids/overexpressing truncated or residue‐altered OTUB1 as shown in Figure 3D. Co‐IP experiment showed that, although the truncated OTUB1 lacking the UBA domain interacted with CCN6, the truncated OTUB1 lacking both UBA and linker domains failed to bind CCN6, indicating that the linker domain is needed for the binding between OTUB1 and CCN6 (Figure 3E).

### 3.4 OTUB1 stabilizes CCN6 through the non‐canonical function

**FIGURE 4:**
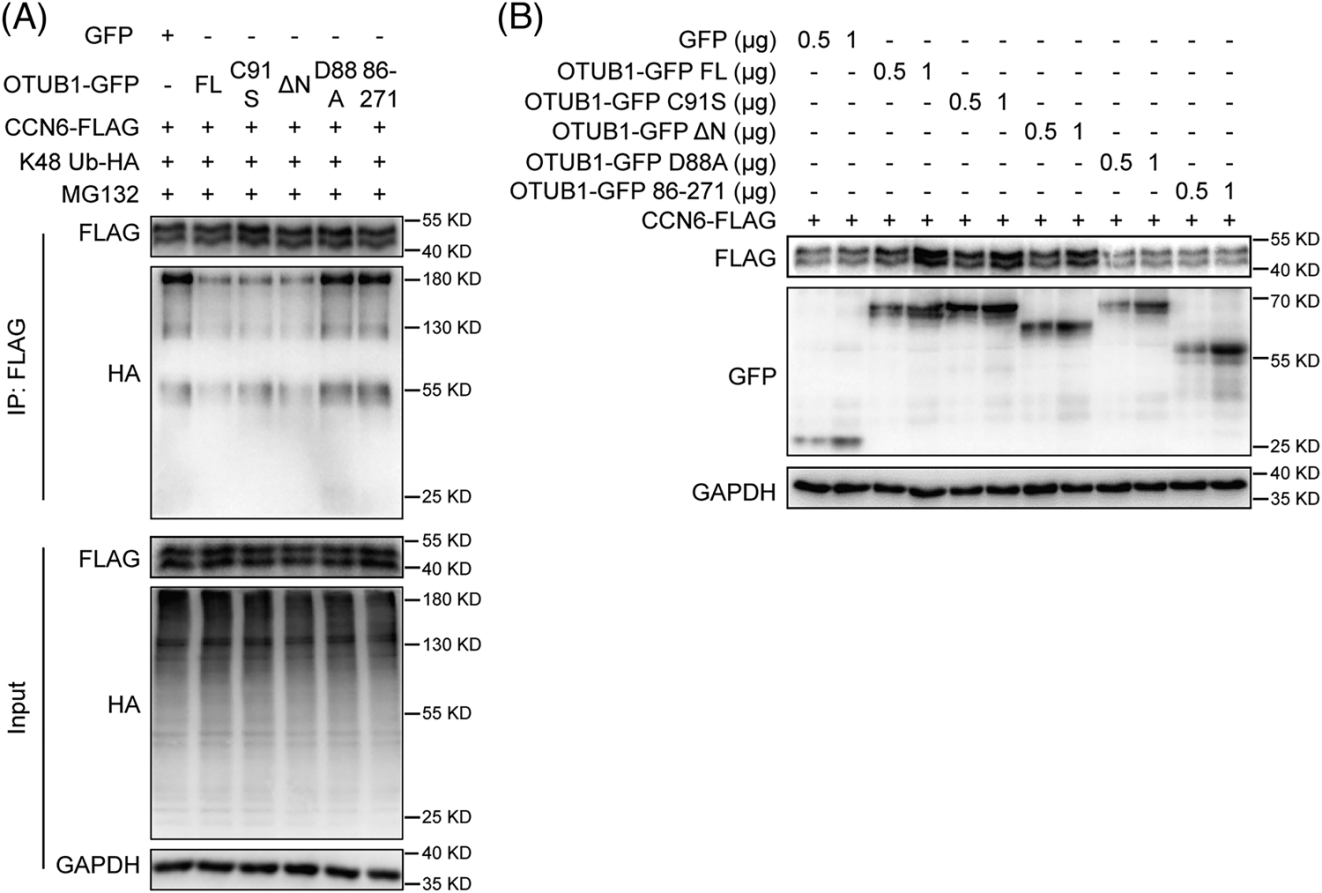
The D88 residue is required for OTUB1 to stabilize CCN6. (A) 4T1 cells were transfected with indicated plasmids. Twenty‐four hours after transfection, cells were treated with MG132 (10 μM) for 6 h before lysation. Thereafter, proteins were immunoprecipitated with anti‐FLAG antibody and analysed with Western blot for FLAG, GFP and GAPDH. (B) 4T1 cells were transfected with indicated plasmids. Twenty‐four hours after transfection, whole‐cell lysates were analysed with Western blot for FLAG, GFP and GAPDH.

Next, we tried to identify the domains or residues of OTUB1 that are essential for CCN6 deubiquitination. K48‐specific ubiquitination of CCN6 was strongly inhibited by full‐length OTUB1 as well as C91S and ∆N OTUB1 mutants. However, the D88A and 86−271 OTUB1 mutants failed to reduce the K48 ubiquitination of CCN6 (Figure 4A and Figure S8). The 86−271 mutant could not deubiquitinate CCN6 due to its inability to interact with CCN6 (Figure 3D). OTUB1 reduces ubiquitination of target proteins by two distinct mechanisms, that is, a canonical way and a non‐canonical manner. The canonical DUB activity enables OTUB1 to directly cleave K48 polyubiquitin chains from its substrates.^22^, ^23^ Alternatively, OTUB1 can also inhibit the ubiquitination of its substrates by obstructing E2/E3‐dependent ubiquitin transfer in a non‐canonical way.^24^, ^25^, ^26^ Of note, the C91 residue is required for the canonical DUB activity of OTUB1, whereas the D88 residue mediates the non‐canonical function of OTUB1.^25^, ^27^, ^28^ The C91S mutant, which lacked the canonical DUB activity, was still able to inhibit the K48 ubiquitination of CCN6 as efficiently as full‐length OTUB1 (Figure 4A). Consistently, we found that OTUB1 could not remove the already synthesized K48 ubiquitin chains on CCN6 in the in vitro deubiquitination assay (Figure S9), indicating that OTUB1 reduces the ubiquitination of CCN6 independent of its canonical activity. However, the D88A mutant lacking non‐canonical function could not reduce the ubiquitination of CCN6, suggesting that OTUB1 inhibits the ubiquitination of CCN6 in a non‐canonical way. Since K48 ubiquitination is linked with 26S proteasome‐mediated protein degradation, both the D88A and 86−271 mutants could not increase CCN6 expression (Figure 4B). Therefore, these findings show that OTUB1 inhibits the K48 ubiquitination and degradation of CCN6 in a non‐canonical way.

### 3.5 OTUB1 inhibits aggressive phenotypes of breast cancer cells by upregulating CCN6

**FIGURE 5:**
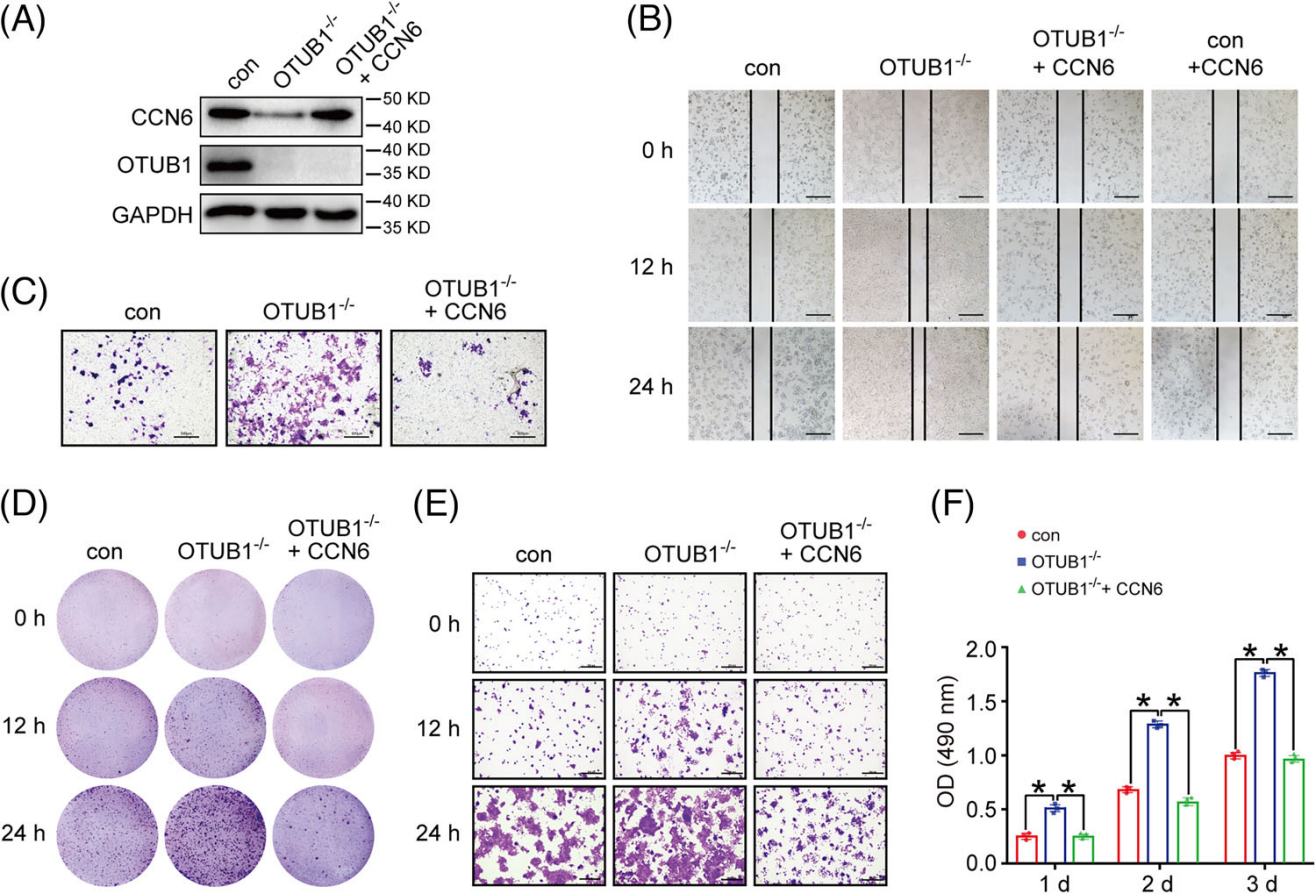
OTUB1 inhibits aggressive phenotypes of breast cancer cells in vitro. (A) Western blot analysis of CCN6, OTUB1 and GAPDH in control, OTUB1^−/−^ and OTUB1^−/−^ + CCN6 4T1 cells. Cell migration of different 4T1 cells was determined by (B) wound healing assay and (C) transwell migration assay (scale bar = 500 μm). Cell proliferation of different 4T1 cells was determined by crystal violet staining. Images were taken with (D) 1× and (E) 4× magnification (scale bar = 500 μm). (F) MTT assays were performed to assess the viability of different 4T1 cells (*n* = 3 for all groups) (mean ± SEM, *p < .05).

Given that CCN6 acts as a suppressor of breast cancer and its protein stability is enhanced by OTUB1, we investigated the impact of OTUB1 on breast cancer cells in vitro. OTUB1‐deficient 4T1 cells migrated faster than control 4T1 cells as determined by the wound healing assay and transwell migration assay (Figure 5A–C and Figure S10A). As shown before, deletion of OTUB1 was accompanied by reduced protein levels of CCN6. To determine whether OTUB1 inhibited 4T1 migration by increasing CCN6, we overexpressed exogenous CCN6 in OTUB1^−/−^ cells (Figure 5A). Interestingly, replenished CCN6 markedly reduced the migration of OTUB1^−/−^ 4T1 cells (Figure 5B,C), indicating that OTUB1 influences cancer cell migration by regulating CCN6. Besides, ablation of OTUB1 significantly increased the proliferation and viability of 4T1 cells, while overexpression of CCN6 in OTUB1^−/−^ cells reversed the effect (Figure 5D–F and Figure S10B), suggesting that OTUB1 inhibits breast cancer proliferation and viability by elevating CCN6 levels. To confirm this finding, we transfected human breast cancer cells with *OTUB1* siRNA. Similar to the findings above, OTUB1 knockdown promoted the migration and proliferation of MDA‐MB‐231 (Figure S11A–F) and BT549 (Figure S11G–L) cells. In addition, we further studied the effect of OTUB1 C91S and D88A mutants on 4T1 cells. As compared with full‐length and C91S OTUB1, the OTUB1 D88A mutant could not efficiently inhibit the proliferation of OTUB1^−/−^ 4T1 cells ( Figure S12A,B), which is in line with the previous finding that the OTUB1 D88A mutant failed to stabilize CCN6 (Figure 4A,B). In aggregate, these results show that OTUB1 inhibits the aggressive phenotypes, including migration, proliferation and viability, of breast cancer cells by upregulating CCN6 protein abundance.

### 3.6 OTUB1 inhibits breast cancer growth in vivo by upregulating CCN6

**FIGURE 6:**
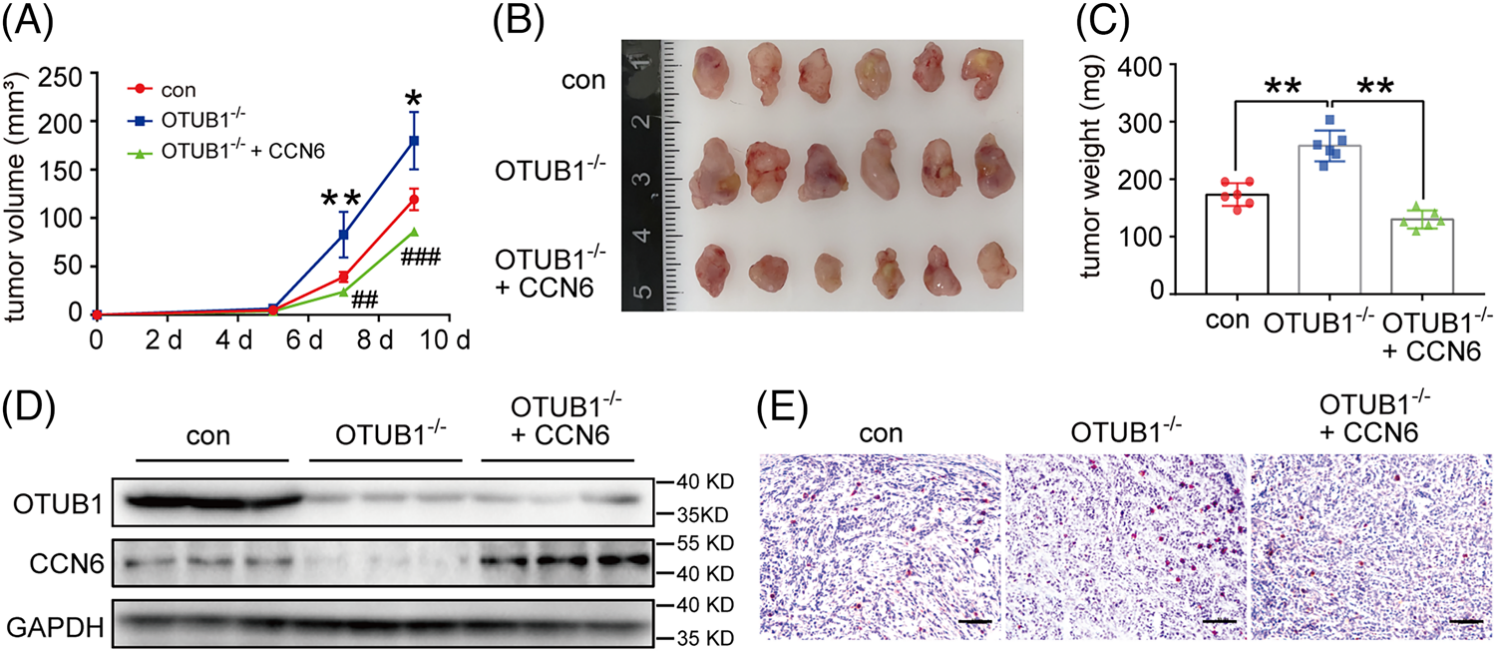
OTUB1 inhibits breast cancer growth in vivo. (A) Control, OTUB1^−/−^ and OTUB1^−/−^ + CCN6 4T1 cells were injected into nude mice and tumour volume was measured (*n* = 6 for all groups) (mean ± SEM, NC vs. OTUB1^−/−^: *p < .05, **p < .01; NC vs. OTUB1^−/−^ + CCN6: ^#^ *p* < .05, ^##^ *p* < .01). On day 9 after tumour inoculation, mice were sacrificed and tumours were collected. Tumour (B) size and (C) weight were measured (*n* = 6 for all groups) (mean ± SEM, *p < .05, **p < .01). (D) Whole‐cell lysates of tumour samples were analysed by Western blot for OTUB1, CCN6 and GAPDH. (E) Tumour samples were stained for Ki‐67 and immunoreactivity was detected by 3,3′‐diaminobenzidine chromogen (brown). Sections were counterstained with haematoxylin (blue) (scale bar = 100 μm).

To study the impact of OTUB1 on breast cancer in vivo, we established the mouse allograft model by subcutaneously injecting control, OTUB1^−/−^ and OTUB1^−/−^ + CCN6 (OTUB1^−/−^ cells transfected with CCN6‐overexpressing plasmids) 4T1 cells in nude mice. The three groups of mice displayed similar body weight changes within 9 days after cancer cell inoculation (Figure S13). Compared with control, the OTUB1^−/−^ group showed significantly increased tumour volume, while the tumour volume was significantly reduced in the OTUB1^−/−^ + CCN6 group (Figure 6A). On day 9 post inoculation, mice were sacrificed and the size and weight of 4T1‐allografted tumours was measured (Figure 6B,C). Similar to Figure 6A, OTUB1 deficiency significantly increased tumour weight whereas supplementation of CCN6 abolished the effect of OTUB1 deletion on tumour weight (Figure 6C), suggesting that OTUB1 inhibits tumour growth primarily by increasing CCN6. In line with in vitro findings, OTUB1‐deficient allografted tumours had reduced levels of CCN6 (Figure 6D). In vitro, OTUB1 4T1 cells were found to proliferate faster than did control cells (Figure 5D–F). To measure cell proliferation in vivo, Ki‐67 expression in isolated tumours was detected by IHC. Consistent with Figure 5D–F, OTUB1‐deficient 4T1‐allografted tumours showed more Ki‐67 positive cells than did control tumours, and Ki‐67 positivity was reduced in tumours upon CCN6 supplementation (Figure 6E). Taken together, these findings demonstrate that OTUB1 suppresses the growth of breast cancer in vivo by increasing the protein levels of CCN6.

### 3.7 OTUB1 expression is positively correlated with CCN6 levels in clinical samples

**FIGURE 7:**
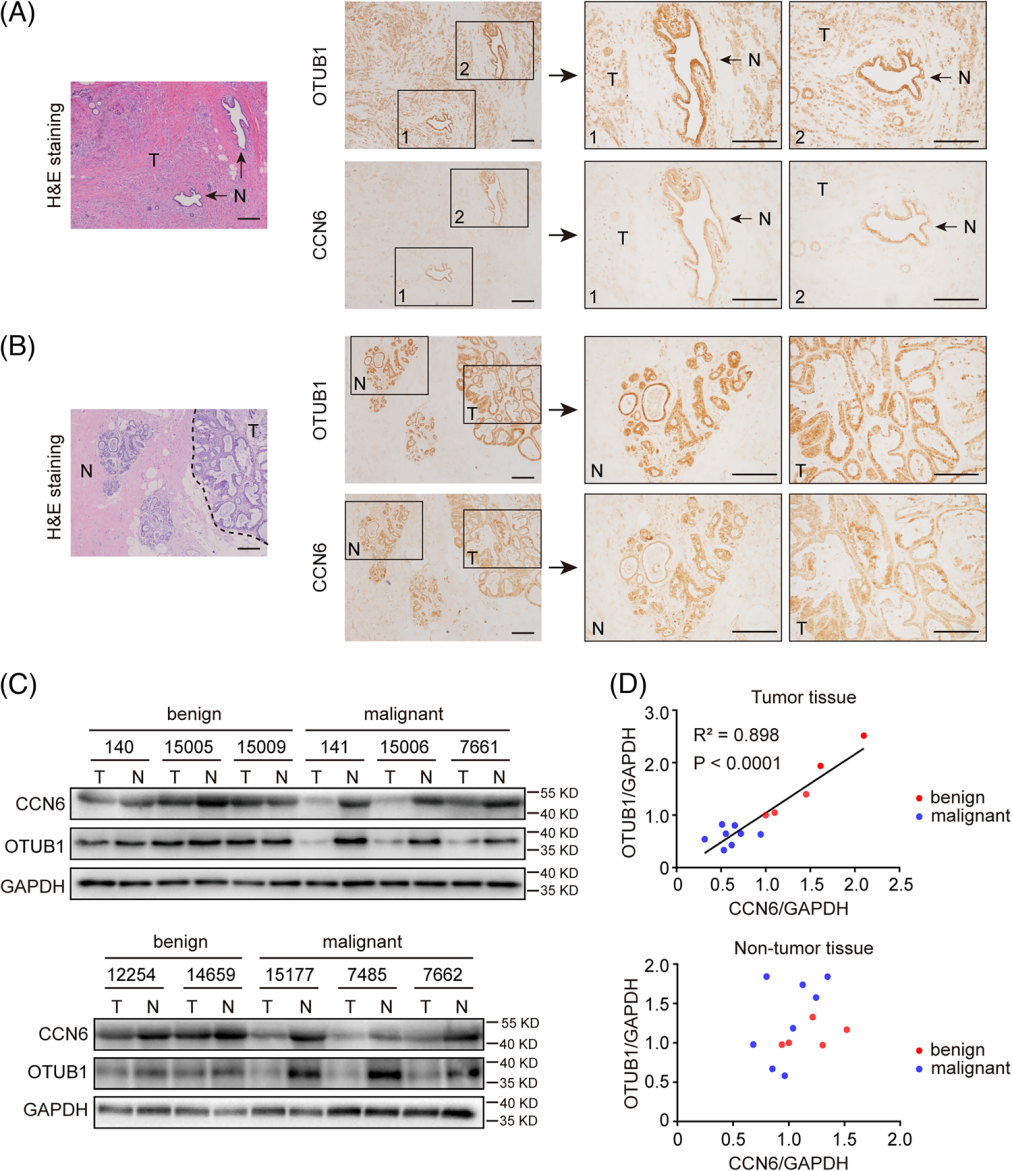
OTUB1 expression is positively correlated with CCN6 in clinical samples. (A) An invasive ductal carcinoma (Grade II, moderately differentiated) sample (T) and the adjacent non‐tumour tissue (N) were analysed with H&E staining and immunohistochemistry for OTUB1 and CCN6 (scale bar = 100 μm). (B) A breast intraductal papilloma sample (T) and the adjacent non‐tumour tissue (N) were analysed with H&E staining and immunohistochemistry for OTUB1 and CCN6 (scale bar = 100 μm). (C) Clinical samples were analysed with Western blot for OTUB1, CCN6 and GAPDH. (D) Correlation between the expression of OTUB1 and CCN6 in tumour tissues (upper panel) and adjacent non‐tumour tissues (lower panel).

To evaluate the clinical significance of OTUB1, tumour tissues and adjacent non‐tumour tissues from patients with breast cancer or benign breast tumour were collected (Table S1). In good agreement with previous reports,^5^, ^11^ we found that CCN6 was downregulated in breast cancer tissues (Figure 7A). Besides, OTUB1 was also strongly reduced in breast cancer tissues as compared to adjacent normal breast tissues (Figure 7A). By contrast, benign breast tumours and adjacent breast tissues showed comparable protein expression of OTUB1 and CCN6 (Figure 7B). To consolidate the findings of the immunohistochemical staining, we further analysed the expression of OTUB1 and CCN6 in clinical samples with Western blot (Figure 7C,D). Malignant tumours had lower levels of OTUB1 and CCN6 than did benign tumours and normal breast tissues, and the levels of OTUB1 were positively correlated with CCN6 levels in breast tumours (Figure 7D). These results show that, clinically, OTUB1 possibly regulates the pathogenesis and development of breast cancer by controlling CCN6 stability.

## 4 DISCUSSION

The turnover of tumour‐suppressing proteins is orchestrated by protein synthesis and degradation, and the enhanced degradation of these proteins might contribute to tumour development and growth. In this study, we found for the first time that the breast cancer suppressor CCN6 was degraded through the UPS. Considering that CCN6 is a secreted protein, it may serve as a valuable biomarker in the non‐invasive diagnosis and prognosis of breast cancer. Intracellular CCN6 is labelled with K48‐linked polyubiquitin chains for recycling by the 26S proteasome, which is critical for regulating CCN6 protein expression. In addition, we identified OTUB1 as a bona fide DUB for CCN6, and OTUB1 played a key role in breast cancer by regulating CCN6 protein levels.

OTUB1 is a unique DUB that has both a canonical deubiquitinating activity and a non‐canonical function. The canonical activity, dependent on the C91 residue, enables OTUB1 to directly and specifically cleave K48‐linked polyubiquitination from substrates.^22^, ^23^ In addition, OTUB1 can interact with E2 ubiquitin‐conjugating enzymes and obstruct ubiquitin transfer to substrates in a non‐canonical way, thereby blocking the synthesis of polyubiquitin chains on target proteins.^24^, ^25^, ^26^, ^29^ Although the canonical catalytic activity of OTUB1 is specific for K48 polyubiquitination, OTUB1 can also reduce other types of ubiquitination, especially K63 ubiquitination, on substrates through the non‐canonical mechanism.^23^, ^26^, ^29^ The non‐canonical function is dependent on the N‐terminal Ub‐binding domain and the D88 residue.^25^, ^27^, ^28^ We found that the D88A mutant, rather than the C91S mutant, of OTUB1 failed to inhibit the ubiquitination and degradation of CCN6, indicating that OTUB1 blocks the synthesis of ubiquitination on CCN6 in a non‐canonical way.

OTUB1 is a DUB belonging to the OTU subfamily. Of note, we and two other groups independently found that global knockout of OTUB1 caused embryonic lethality in mice.^30^, ^31^, ^32^ Using tissue‐specific OTUB1 knockout mice, we and others have shown that OTUB1 is involved in CNS autoimmunity,^31^ lupus‐like autoimmunity,^33^ sepsis,^34^ liver infection^35^ and T cell response^36^ by regulating astrocytes, B cells, dendritic cells, hepatocytes and T cells, respectively.

In addition to immune regulation, OTUB1 has emerged as an important modulator in cancer. OTUB1 has been shown to promote bladder cancer,^37^ colorectal cancer,^38^ oesophageal cancer,^39^ lung cancer^40^ and multiple myeloma.^41^ However, OTUB1 can also act as a tumour suppressor, since it increases the stability and activity of p53.^27^ Using inducible OTUB1 KO mice, Zhou et al. show that deletion of OTUB1 profoundly boosts anticancer immunity by increasing the activity of NK cells and CD8^+^ T cells.^36^ The carcinogenesis is a complex process and it is regulated by both oncogenes and tumour suppressor genes. A recent study shows that OTUB1 contributes to breast tumourigenesis by stabilizing MYC.^42^ Similar to p53, MYC functions in many kinds of cancers and is not specific to breast cancer.^43^, ^44^ Given that (i) OTUB1 stabilizes p53,^27^, ^45^ (ii) MYC can also be stabilized by OTUB1^42^ and (iii) p53 is a tumour‐suppressing protein while MYC is an oncoprotein,^43^, ^44^ the role of OTUB1 in breast cancer remains elusive. In this study, we found that OTUB1 inhibited the aggressive phenotypes of breast cancer by stabilizing CCN6, a specific and potent suppressor in breast cancer.^4^, ^5^, ^6^ Deletion of OTUB1, accompanied by reduced CCN6 protein abundance, enhanced the growth of 4T1 cells in vitro and in vivo, and the effect of OTUB1 deletion was abolished by CCN6 overexpression, unambiguously showing that OTUB1 inhibits breast tumour via stabilizing CCN6. However, considering the discrepancies between former reports and the present study, the clinical role of OTUB1 in breast cancer should be treated with more caution and further clinical studies are definitely required.

Previous studies have found that CCN6 expression is reduced in aggressive breast cancer.^5^, ^11^ Consistently, we detected reduced protein levels of OTUB1 and CCN6 in malignant breast cancer tissues as compared to non‐tumorous breast tissues and benign tumours, indicating that the reduced CCN6 protein expression in aggressive breast cancer should be, at least partially, attributed to the altered OTUB1 expression. OTUB1 mRNA levels were not significantly correlated with the survival time of patients with breast cancer.^47^ Our finding that the protein levels of OTUB1 and CCN6 are reduced in aggressive breast cancer suggests that the protein levels of OTUB1 in cancer tissues are more valuable for the prognosis of breast cancer.

In addition to cytosolic proteins, secreted proteins are also regulated by the UPS. Newly synthesized secreted proteins and membrane‐bound proteins are transported into the lumen of endoplasmic reticulum (ER).^48^ Proteins that do not pass the quality control in the ER retrotranslocate into the cytosol, where they are ubiquitinated for degradation. This process, called ERAD, is essential for the quality control of secreted proteins.^49^ In addition to misfolded proteins, nascent secreted proteins can also be degraded via the UPS.^50^ In this study, we found that OTUB1 prevented the proteasomal degradation of the secreted protein CCN6 by reducing its K48 ubiquitination, showing for the first time that OTUB1 can regulate secreted proteins. Nascent functional CCN6 retrotranslocates to the cytoplasm for ubiquitination and degradation, which may serve as a post‐translational mechanism controlling the balanced production of secreted proteins.

The OTU subfamily of DUBs is composed of 17 members and many members are involved in breast cancer regulation. OTULIN,^51^ ZRANB1,^52^ A20^17^ and OTUD7B^53^ have been shown to promote breast cancer, whereas OTUD1,^18^ OTUD3^19^ and OTUD4^54^ play a suppressive role in breast cancer. In this study, we identified OTUB1 as a new regulator of breast cancer by stabilizing the tumour suppressor CCN6. These findings collectively suggest that DUBs of the OTU family may become potential therapeutic targets for breast cancer.

## CONFLICT OF INTEREST STATEMENT

The authors declare no conflicts of interest.

## Supporting information

Supporting Information
