# Supplementary material for: OTUB1 inhibits breast cancer by non‐canonically stabilizing CCN6
Source: Clin Transl Med. 2023 Aug 22;13(8):e1385. doi: 10.1002/ctm2.1385 (PMC10444971; doi:10.1002/ctm2.1385)
Supplement: Supplementary file 1 — Supporting Information [file CTM2-13-e1385-s001.docx]

***Supplementary Information***

**OTUB1 inhibits breast cancer by non-canonically stabilizing CCN6**

The supplementary file includes 1 Table and 13 Figures.

**Supplementary Table 1.** Information of patients

| Nr. | Sex | Age | Lesion | Pathological diagnosis | Sentinel lymph node metastasis |
| --- | --- | --- | --- | --- | --- |
| F22-00140 | Female | 44 | Left breast | Breast fibroadenoma (Benign) | No |
| F21-15005 | Female | 54 | Right breast | Breast adenopathy with intraductal papilloma (Benign) | No |
| F21-15009 | Female | 24 | Right breast | Breast fibroadenoma (Benign) | No |
| F22-00141 | Female | 40 | Right breast | Invasive ductal carcinoma (Grade II, moderately differentiated) | No |
| F21-15006 | Female | 54 | Left breast | Invasive ductal carcinoma (Grade II, moderately differentiated) | Yes |
| F21-07661 | Female | 69 | Left breast | Invasive ductal carcinoma (Grade III, poorly differentiated) | No |
| F21-12254 | Female | 45 | Right breast | Breast fibroadenoma (Benign) | No |
| F21-14659 | Female | 58 | Left breast | Benign phyllodes tumor (Benign) | No |
| F21-15177 | Female | 49 | Left breast | Invasive ductal carcinoma (Grade II, moderately differentiated) | Yes |
| F21-07485 | Female | 70 | Right breast | Invasive ductal carcinoma (Grade II, moderately differentiated) | Yes |
| F21-07662 | Female | 63 | Left breast | Invasive ductal carcinoma (Grade III, poorly differentiated) | No |
| F21-14996 | Female | 50 | Right breast | Invasive ductal carcinoma (Grade II, moderately differentiated) | No |
| F21-11909 | Female | 53 | Left breast | Invasive ductal carcinoma (Grade III, poorly differentiated) | No |


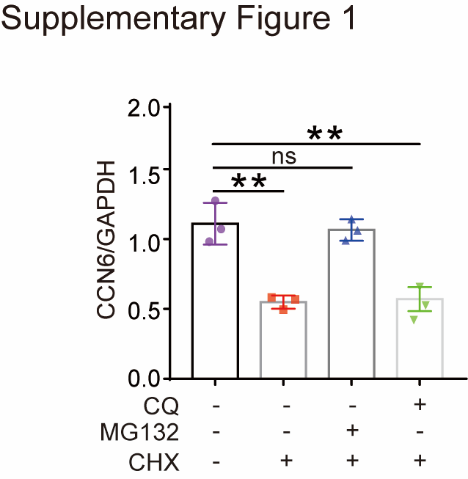


The relative protein levels of CCN6 normalized to GAPDH (n = 3 per group) (mean + SEM, * *p* < 0.05, ** *p* < 0.01).


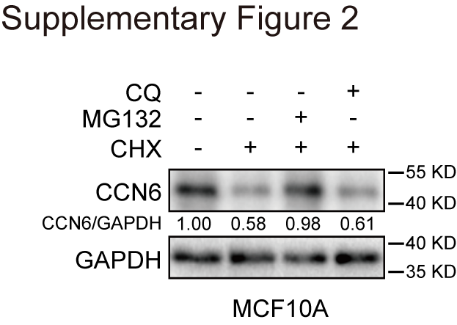


MCF10A cells were left untreated or treated with cycloheximide (CHX, 100 μg/ml), MG132 (10 μM), and CQ (50 μM), either alone or in combination as indicated, for 9 hours. Thereafter, CCN6 protein abundance was analyzed by western blot.


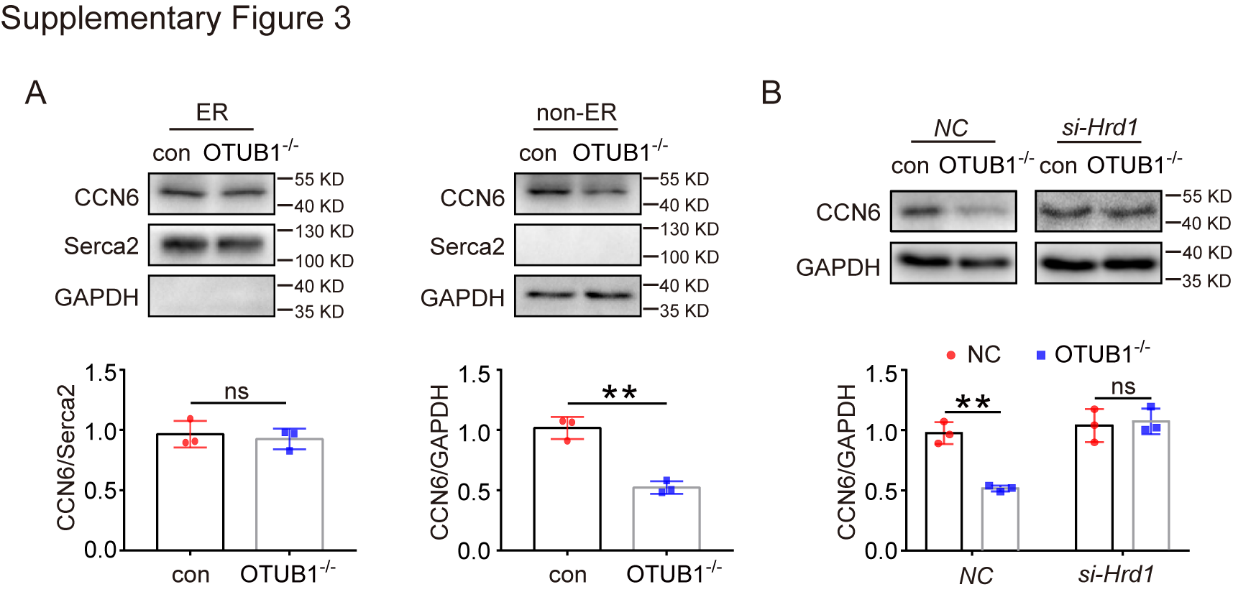


(A) Endoplasmic reticulum and non-endoplasmic reticulum fractions were extracted from the control and OTUB1^-/-^ 4T1 cells. CCN6 levels were measured by western blot (upper panel). The lower panel shows the quantification (mean + SEM, * *p* < 0.05, ** *p* < 0.01).

(B) Control and OTUB1^-/-^ 4T1 cells were transfected with nonsense or *Hrd1* siRNA for 48 hours. The protein abundance of CCN6 was detected by western blot (upper panel). The lower panel shows the quantification (mean + SEM, * *p* < 0.05, ** *p* < 0.01).


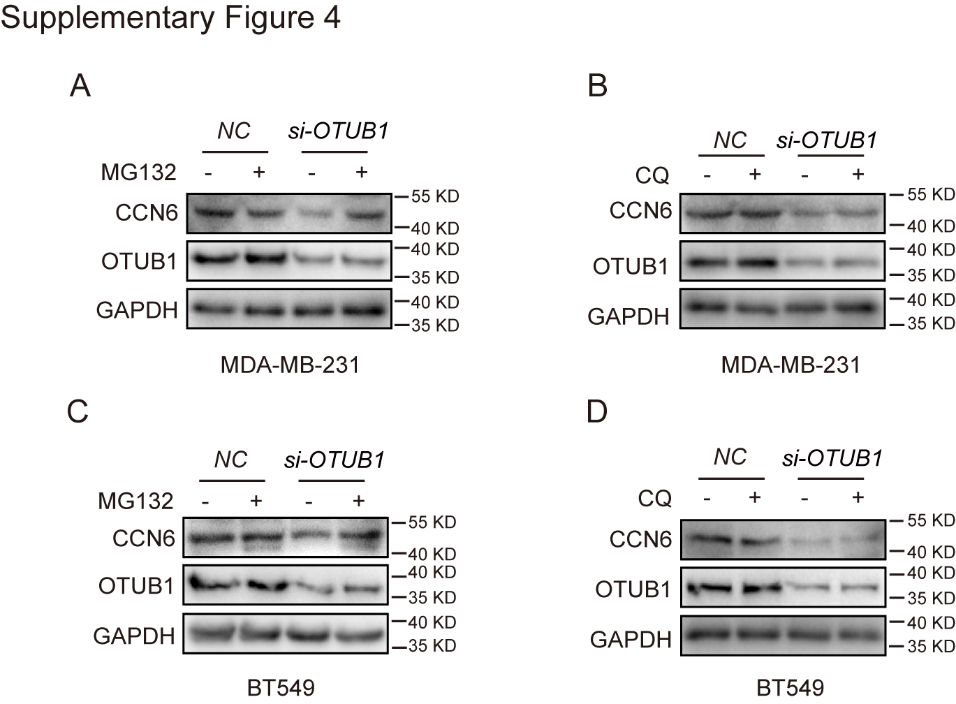


(A-B) MDA-MB-231 cells were transfected with nonsense siRNA or *OTUB1* siRNA. 48 hours after transfection, cells were treated with (A) 10 μM MG132 or (B) 50 μM CQ for 0 and 6 hours. Thereafter, whole-cell lysates were analyzed by western blot.

(C-D) BT549 cells were transfected with nonsense siRNA or *OTUB1* siRNA. 48 hours after transfection, cells were treated with (C) 10 μM MG132 or (D) 50 μM CQ for 0 and 6 hours. Thereafter, whole-cell lysates were analyzed by western blot.


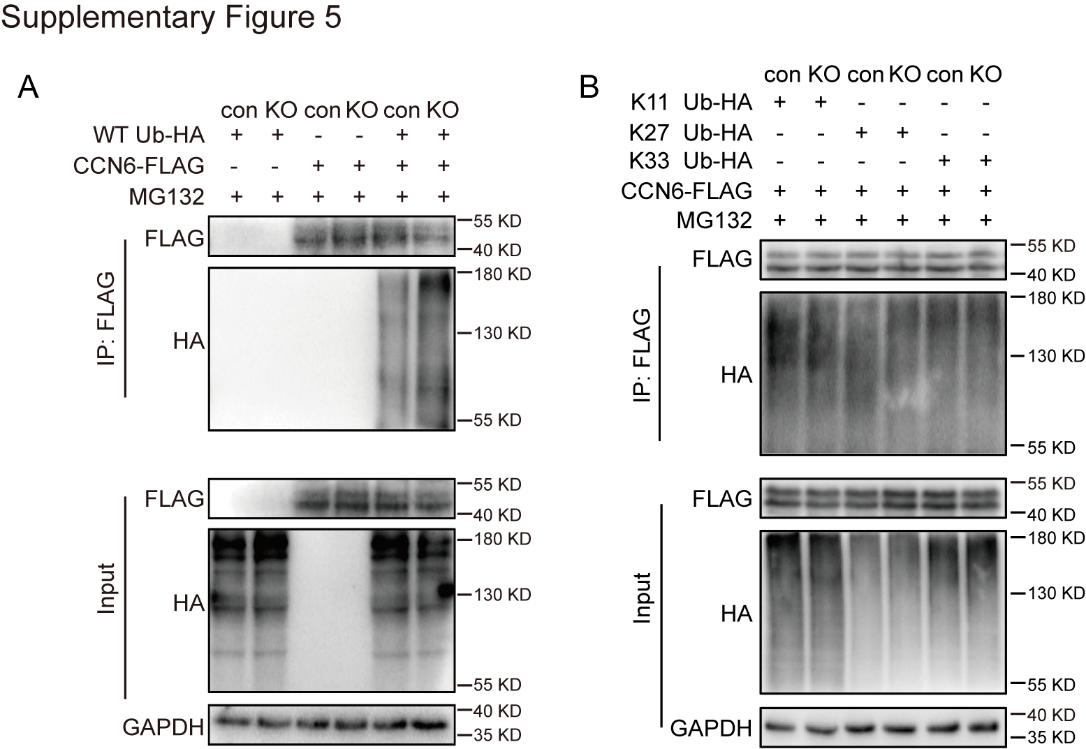


(A-B) Control and OTUB1^-/-^ 4T1 cells transfected with indicated plasmids were treated with MG132 (10 μM) for 6 h before harvest. Proteins were immunoprecipitated with anti-FLAG antibody and analyzed by western blot with indicated antibodies.


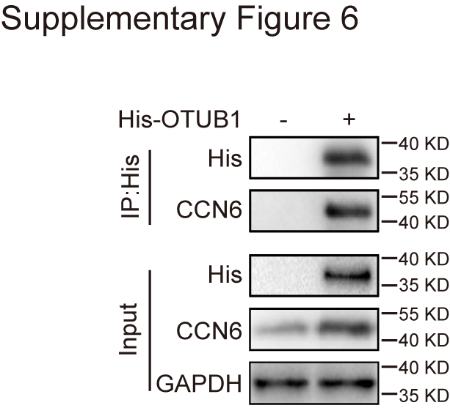


Recombinant His-OTUB1 was incubated with 4T1 cell lysates overnight. Proteins were then immunoprecipitated with anti-His antibody and detected by western blot with indicated antibodies.


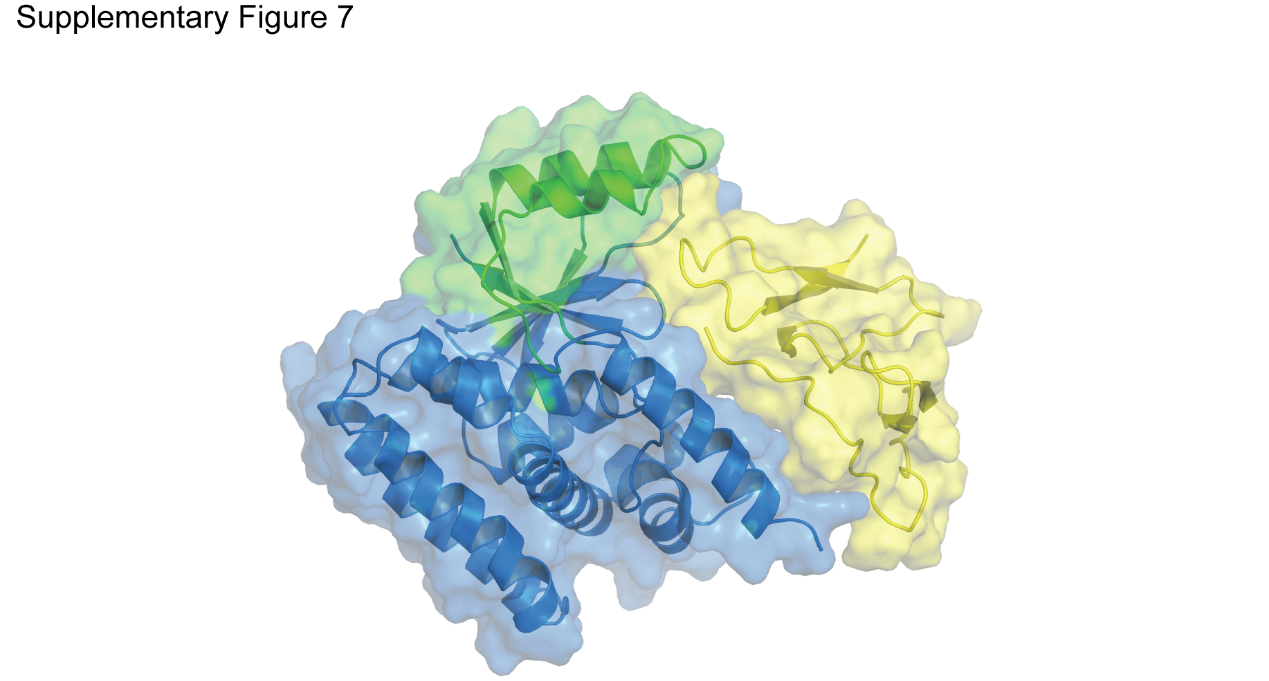


The predicted binding mode of OTUB1 and CCN6. OTUB1 is in blue and the linker domain of OTUB1 is in green. CCN6 is in yellow.


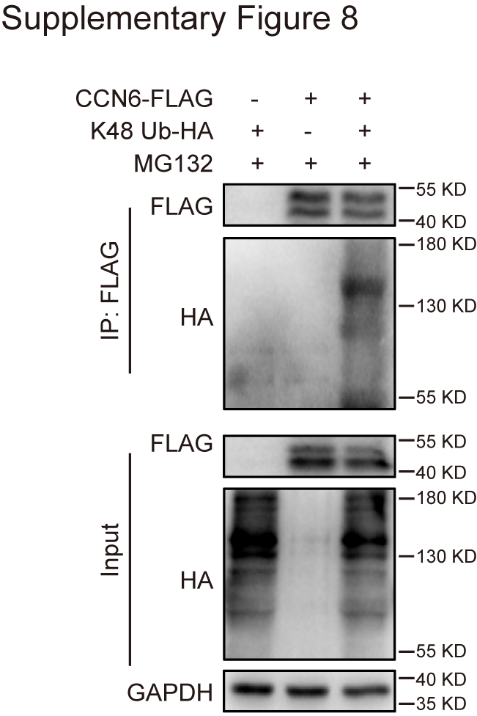


4T1 cells transfected with indicated plasmids were treated with MG132 (10 μM) for 6 h before harvest. Thereafter, proteins were immunoprecipitated with anti-FLAG antibody and analyzed by western blot for FLAG, HA and GAPDH.


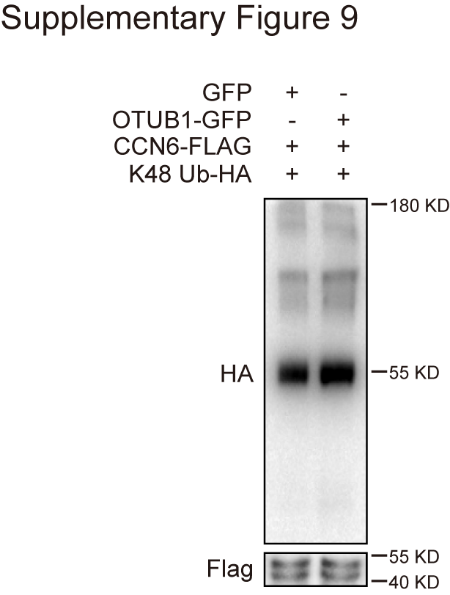


4T1 cells transfected with K48 Ub-HA and CCN6-FLAG were treated with MG132 (10 μM) for 6 hours before harvest. Twenty-four hours after transfection, K48 ubiquitinated CCN6 was harvested by immunoprecipitation with anti-FLAG antibody and incubated with GFP or OTUB1-GFP. After incubation, the mixture was analyzed by western blot for K48 ubiquitination.


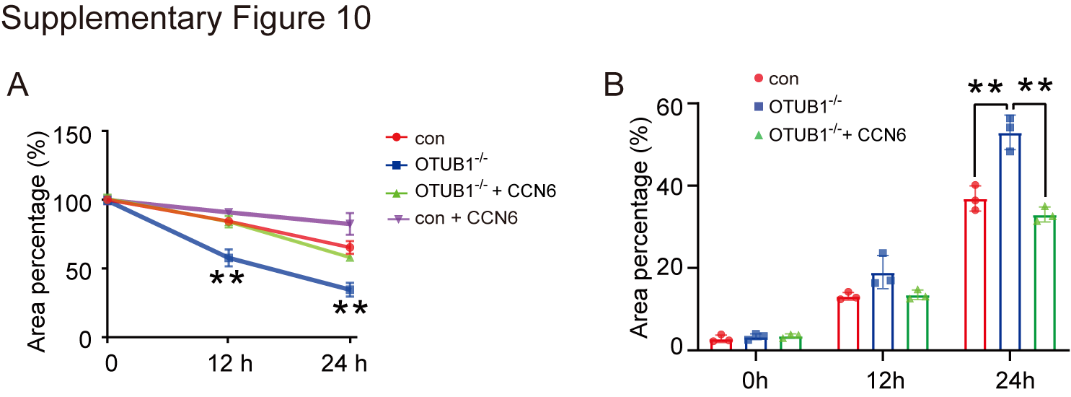


(A) Quantification of the wound healing assay (n = 3 for all groups) (mean + SEM, ** *p* < 0.01).

(B) Quantification of the proliferation assay (n = 3 for all groups) (mean + SEM, ** *p* < 0.01).


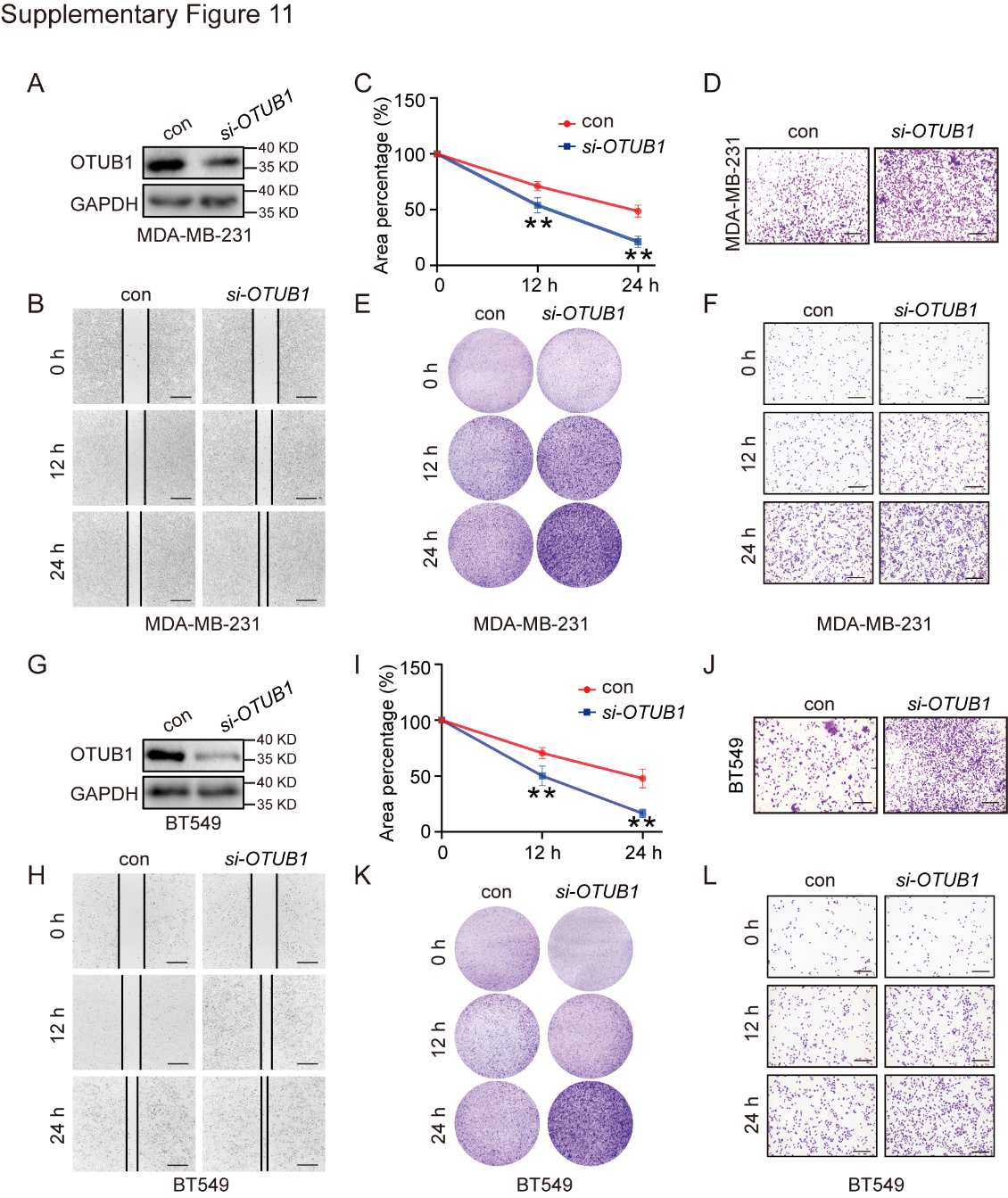


(A) The knockout rate of OTUB1 in MDA-MB-231 cells was determined by western blot.

(B-D) Cell migration of transfected with nonsense siRNA or *OTUB1* siRNA MDA-MB-231 cells was determined by (B-C) wound healing assay and (D) transwell migration assay (scale bar = 500 µm) (mean + SEM, ** *p* < 0.01).

(E-F) Cell proliferation of transfected with nonsense siRNA or *OTUB1* siRNA MDA-MB-231 cells was determined by crystal violet staining. Images were taken with (E) 1× and (F) 4× magnification (scale bar = 500 µm).

(G) The knockout rate of OTUB1 in BT549 cells was determined by western blot.

(H-K) Cell migration of transfected with nonsense siRNA or *OTUB1* siRNA BT549 cells was determined by (H-I) wound healing assay and (K) transwell migration assay (scale bar = 500 µm) (mean + SEM, ** *p* < 0.01).

(J-L) Cell proliferation of transfected with nonsense siRNA or *OTUB1* siRNA BT549 cells was determined by crystal violet staining. Images were taken with (J) 1× and (L) 4× magnification (scale bar = 500 µm).


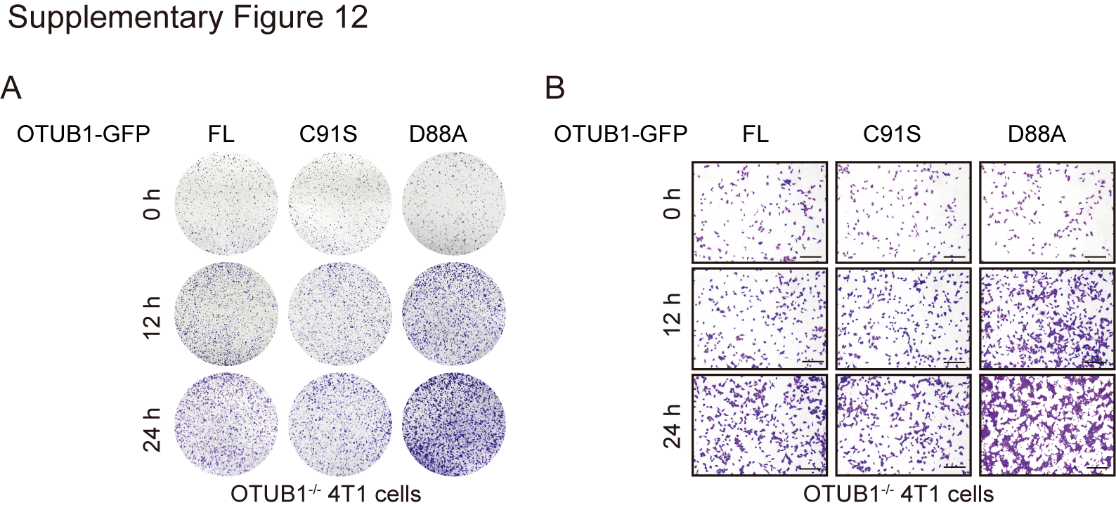


(A-B) OTUB1^-/-^ 4T1 cells were transfected with GFP-OTUB1 FL, GFP-OTUB1 C91S and GFP-OTUB1 D88A plasmids. Cell proliferation was determined by crystal violet staining. Images were taken with (E) 1× and (F) 4× magnification (scale bar = 500 µm).


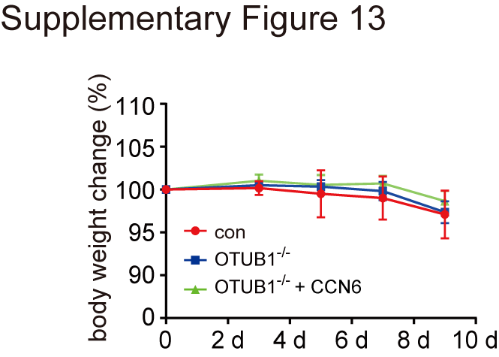


Control, OTUB1^-/-^, and OTUB1^-/-^ + CCN6 4T1 cells were injected subcutaneously into nude mice. The body weight was measured (n = 6 for all groups).
